# Evaluation of Prosthetic Outcomes and Patient Satisfaction With 3D-Printed Implant-Supported Fixed Prosthesis

**DOI:** 10.7759/cureus.42537

**Published:** 2023-07-27

**Authors:** Christian Brenes, Sompop Bencharit, Taylor Fox

**Affiliations:** 1 Prosthodontics, Medical University of South Carolina, Charleston, USA; 2 Digital Dentistry, Medical University of South Carolina, Charleston, USA

**Keywords:** dental 3d printed resin, 3d printed implant prosthesis, patient satisfaction edentulous patients, 3d printed prosthesis, implant-supported prosthesis

## Abstract

Objectives: The objectives of this study were to quantify the number and type of prosthetic complications associated with 3D-printed implant-supported fixed prostheses (3DISFP) and to evaluate patient satisfaction and oral health-related quality of life over a four-month period.

Methods: Fifteen edentulous patients who underwent implant therapy were included in the study. Each patient received a 3D-printed prosthesis using OnX dental resin. Prosthetic complications were documented, and data from the 14-item Oral Health Impact Profile (OHIP) questionnaire were collected at two time points: at enrollment and during a four-month recall.

Results: During the four-month evaluation period, a total of nine complications were recorded, with three classified as catastrophic failures. Statistical analysis revealed statistically significant differences in OHIP scores between the preoperative and postoperative assessments (p<0.001).

Conclusion: Within the limitations of this study, it can be concluded that utilizing 3D-printed prostheses with OnX resin represents a viable alternative for long-term implant-supported temporaries. The patients experienced a significant improvement in their oral health-related quality of life. These results suggest that 3D printing technology, combined with the use of OnX resin, holds promise in providing satisfactory clinical outcomes and enhanced patient satisfaction. However, it is important to acknowledge the limitations of this study, and further research is warranted to validate these findings and explore the long-term performance and durability of 3D-printed implant-supported fixed prostheses. This study contributes to the growing body of evidence supporting the effectiveness of 3D printing technology in implant dentistry. The results highlight the potential of 3DISFP with OnX resin to improve oral health-related quality of life in edentulous patients. Continued advancements in 3D printing materials and techniques will likely expand the utilization of these prostheses, ultimately benefiting patients in need of implant-supported restorations.

## Introduction

Edentulism, the complete loss of natural teeth, affects millions of people worldwide, with approximately 36 million adults in the United States alone being completely edentulous [1]. This condition has a significant impact on oral health and overall quality of life, leading to difficulties in speaking, chewing, and swallowing, as well as facial structure and esthetic changes. Additionally, edentulism has been linked to various systemic health conditions, including cardiovascular and gastrointestinal diseases, diabetes, and malnutrition [2]. Traditional removable complete dentures have been commonly used to address the challenges in losing oral masticatory function posed by edentulism, but conventional complete dentures often fall short, introducing a new set of common removable denture problems such as poor retention, instability, and decreased patient satisfaction [3]. The instability of dentures is a frequent complaint among patients, and the high rate of annual bone resorption observed in the edentulous arches further exacerbates the situation [4,5]. Fixed implant prostheses eliminate several issues related to removable prostheses; however, the cost of implant surgery together with laboratory expenses for prostheses prevent most edentulous patients to have this option.

In recent years, the utilization of additive manufacturing or 3D printing technology has gained momentum in the fabrication of dental prostheses, offering a more affordable solution compared to conventional materials such as metal-acrylic or zirconia, while maintaining accuracy and efficiency in the method of fabrication [6]. Specific to dentistry, 3D printing and subtractive manufacturing or milling are the primary computer-assisted manufacturing (CAM) technologies employed for prosthetic fabrication. While both methods have their advantages and disadvantages, the affordability of technology implementation and fabrication are crucial considerations for dental practices. Generally, 3D printing is regarded as more affordable than milling due to lower initial investment requirements, as affordable 3D printing became a reality in the last decade. Moreover, the fabrication process is typically less labor-intensive compared to milling. The milling requires a higher volume of material compared to 3D printing [7,8]. Furthermore, a recent study investigated the cost-effectiveness of in-house 3D printing in areas such as orthopedics and maxillofacial surgery. The study demonstrated that 3D printing was more cost-effective, primarily due to the lower cost of materials and the ability to produce multiple prostheses simultaneously [9].

Despite the potential cost savings and other advantages, there is limited information available on the clinical performance of 3D-printed biomaterials. Historically, metal-acrylic and zirconia have been the primary biomaterials used for implant-supported fixed prostheses or hybrid prostheses due to their mechanical properties and biocompatibility [10]. Multiple studies have showcased that these properties may contribute to long-term durability and success in clinical settings [11-13]. On the other hand, 3D-printed resins, particularly urethane methacrylate-based resins, have emerged as a promising alternative for dental prostheses. These resins offer high accuracy and precision, with the added advantage of the ability to manufacture complex geometries that are often challenging to produce with milling technologies or even conventional denture processing. In addition, another clinical advantage is that because of the lower cost of the 3D printers compared to milling machines, many dental practices already have 3D printers, whereas milling machines may not be as common [14].

A recent study comparing the longevity of milled zirconia restorations to 3D-printed resin restorations found similar survival rates over a two-year period for implant-supported prostheses. The study concluded that 3D-printed resins can serve as a suitable alternative to milled zirconia, but further long-term studies are necessary to evaluate their performance in clinical settings [13]. Currently, advancements in dental 3D printing are predominantly focused on developing new biocompatible materials with enhanced properties. For instance, Sprintray OnX resin (Sprintray, California, USA), a radiopaque nanoceramic resin introduced in 2021, is specifically designed for 3D printing denture teeth and provisional prosthetic restorations [14]. This resin incorporates a high concentration of ceramic component and is formulated to optimize mechanical properties. The objective of this study was to investigate clinical complications associated with 3D-printed prostheses over a four-month period while assessing patient satisfaction using the Oral Health Impact Profile-14 (OHIP-14), a widely accepted measurement tool for oral health-related quality of life [15].

## Materials and methods

A total of 15 patients seeking treatment were enrolled in the study at the Medical University of South Carolina (MUSC) Digital Dentistry Master program. These patients had previously undergone implant therapy, with a minimum of four osseo-integrated implants uncovered, enabling two clinicians to restore them using either a single or bimaxillary fixed implant-supported prosthesis. As part of the evaluation process, all patients completed an initial OHIP-14 questionnaire. The study protocol was reviewed and approved for exemption by MUSC Institutional Review Board for Human Research, IRB# Pro00118179.

**Figure 1:**
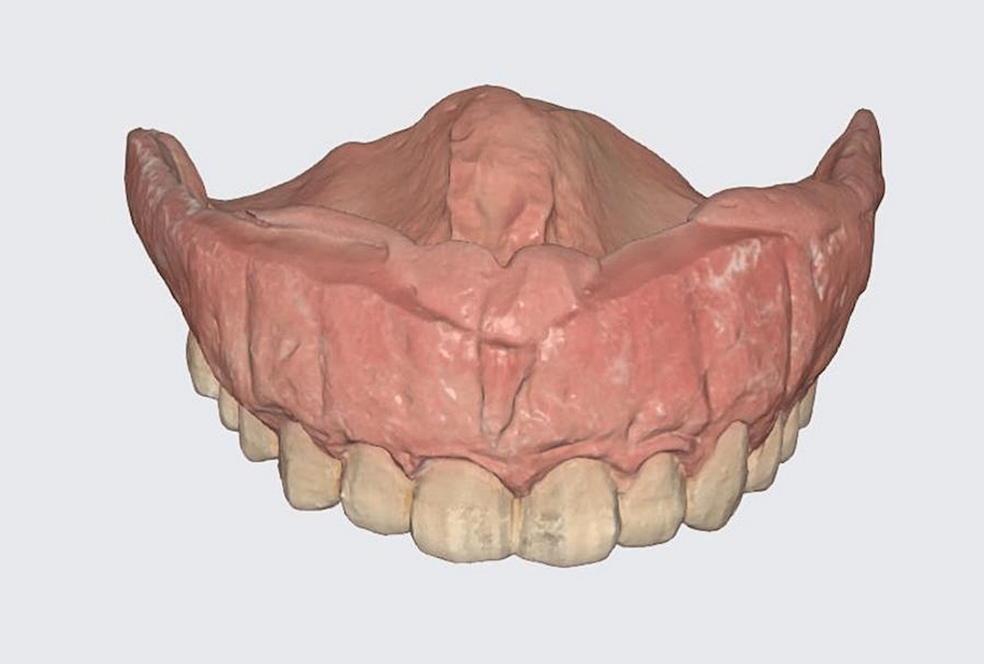
Denture replica scan in color after relining.

**Figure 2:**
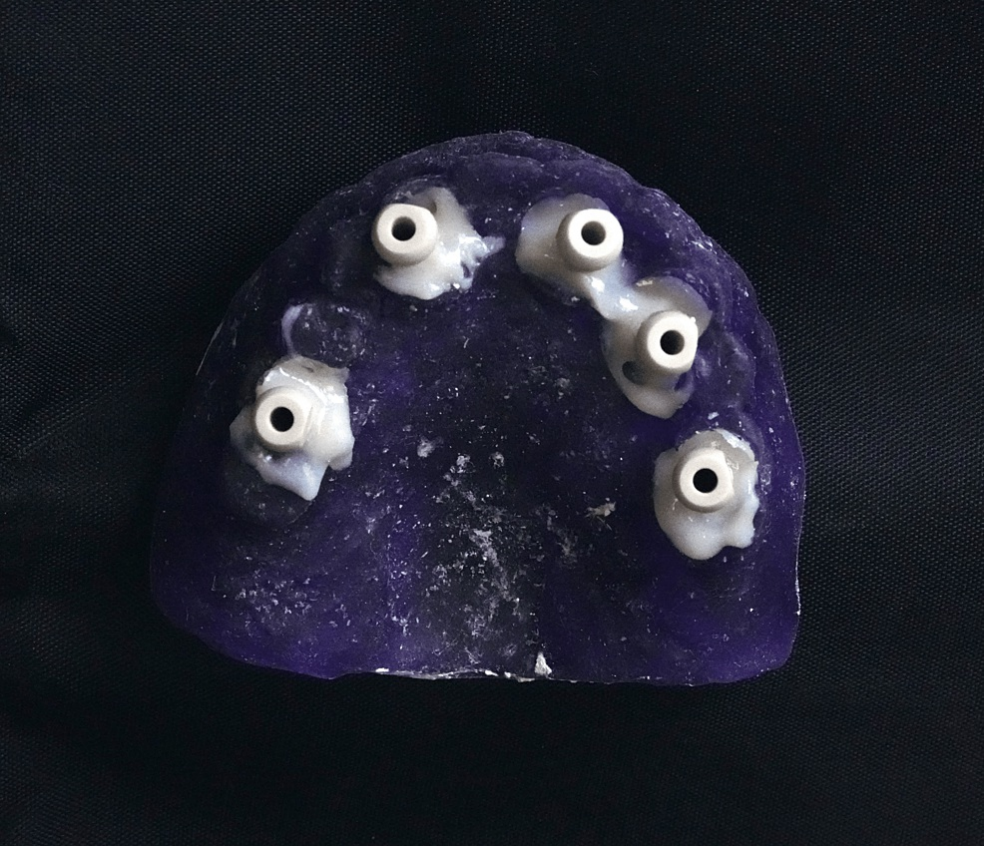
Custom-printed tray used to pick up scan bodies in position.

In this study, a 3D-printed replica of the interim denture or scan appliance was employed as a "prosthetic guide." This guide facilitated the precise placement of scan bodies for scanning purposes, using the Medit T710 laboratory scanner (Medit Corp, Seoul, South Korea), and enabled the generation of a passivity verification model (Figures 1, 2).

**Figure 3:**
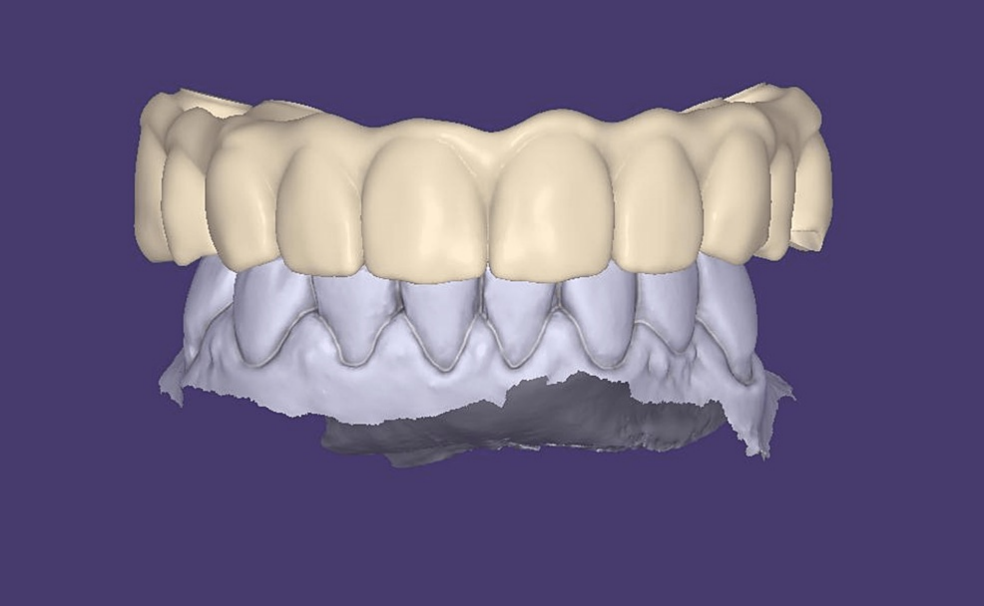
Implant-supported fixed prosthesis designed in exocad.

For data collection, intraoral scans of the denture, opposing arch, and maxilla-mandibular relationship were captured using the Medit i700W (Medit Corp, Seoul, South Korea) and Trios 4 (3Shape, Copenhagen, Denmark) intraoral scanners. These digital records were utilized in the design of the hybrid prosthesis using exocad software (exocad GmbH, Darmstadt, Germany) (Figure 3).

**Figure 4:**
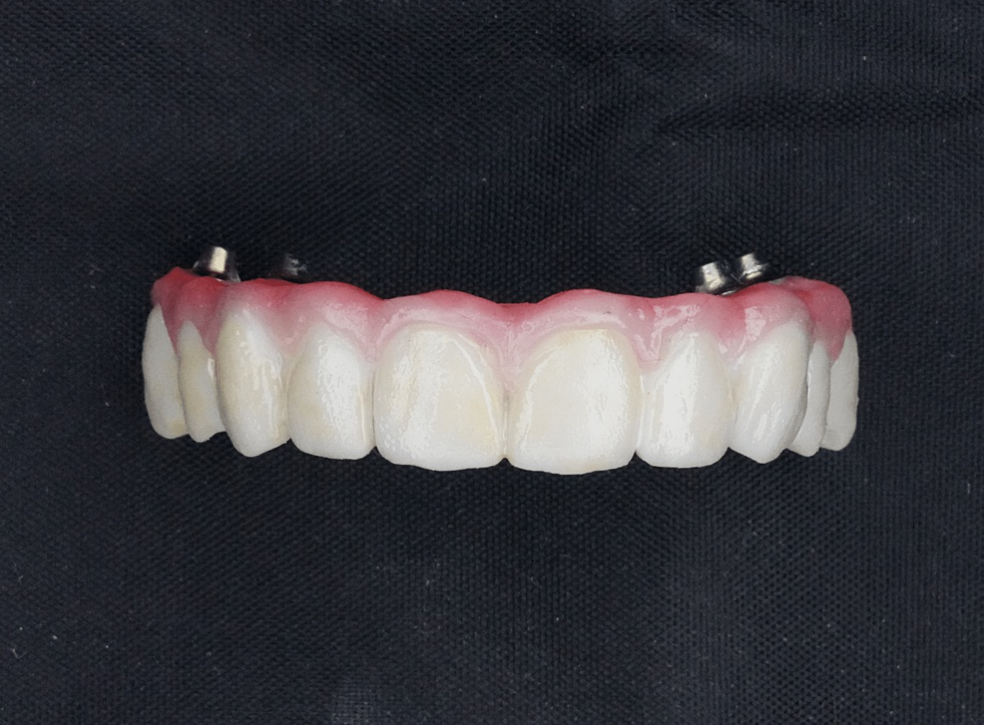
Frontal view of OnX 3D-printed implant-supported prosthesis.

**Figure 5:**
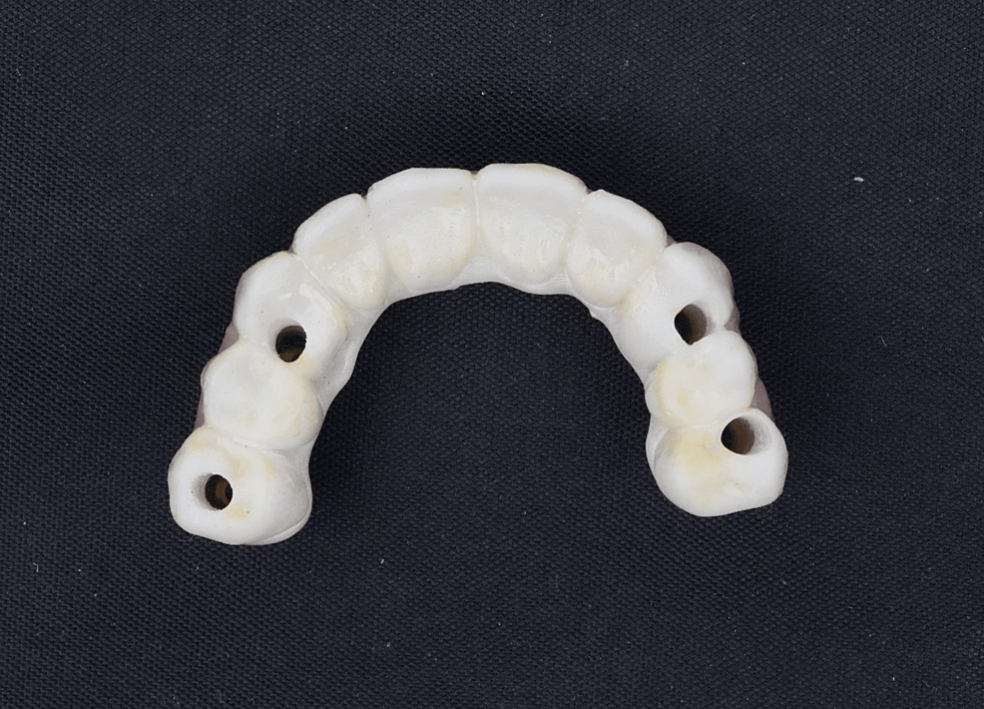
Occlusal view of OnX 3D-printed implant-supported prosthesis.

Subsequently, all prostheses were 3D printed using the Sprintray Pro 55 3D printer and OnX resin (Sprintray, California USA), following the recommended post-curing process. The final step involved post-curing with the Sprintray Procure 2 device. To secure the titanium bases cement, Panavia SA universal resin cement (Kuraray, Chiyoda City, Tokyo, Japan) was used, and gingival characterization, along with polishing and glazing, was performed prior to delivery (Figures 4, 5).

**Figure 6:**
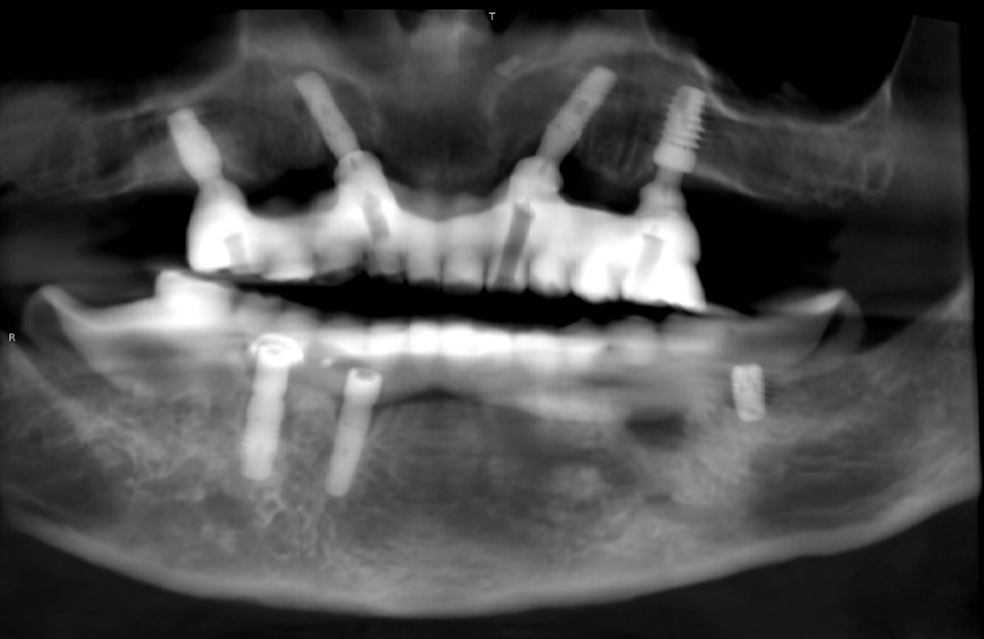
Panoramic reconstruction of the CBCT image at delivery for evaluation of fit of OnX implant-supported prosthesis. CBCT, cone beam computed tomography.

**Figure 7:**
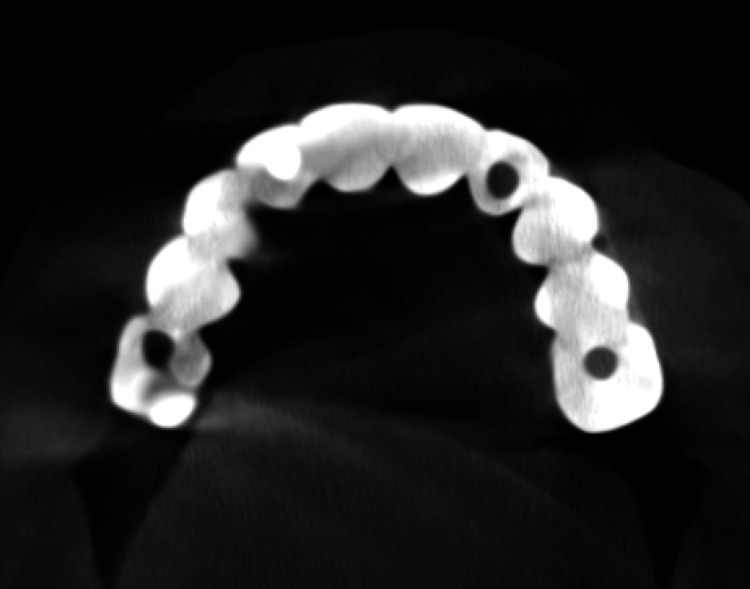
Occlusal view of the radiographic image of the prosthesis at delivery for evaluation of OnX implant-supported prosthesis.

**Figure 8:**
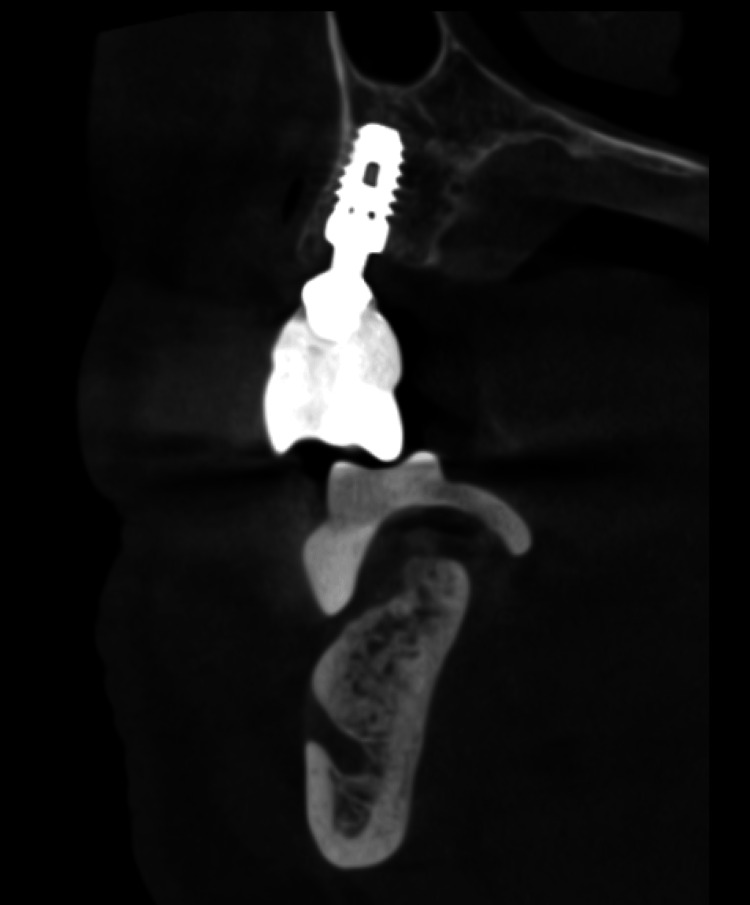
Sagittal view of the radiographic image of the prosthesis at delivery for evaluation of OnX implant-supported prosthesis.

Upon delivery, all prostheses underwent a clinical evaluation to assess their passive fit, achieve ideal occlusion, and ensure satisfactory esthetics. The use of OnX resin facilitated radiographic evaluation due to its radio-opaque properties (Figures 6-8).

**Figure 9:**
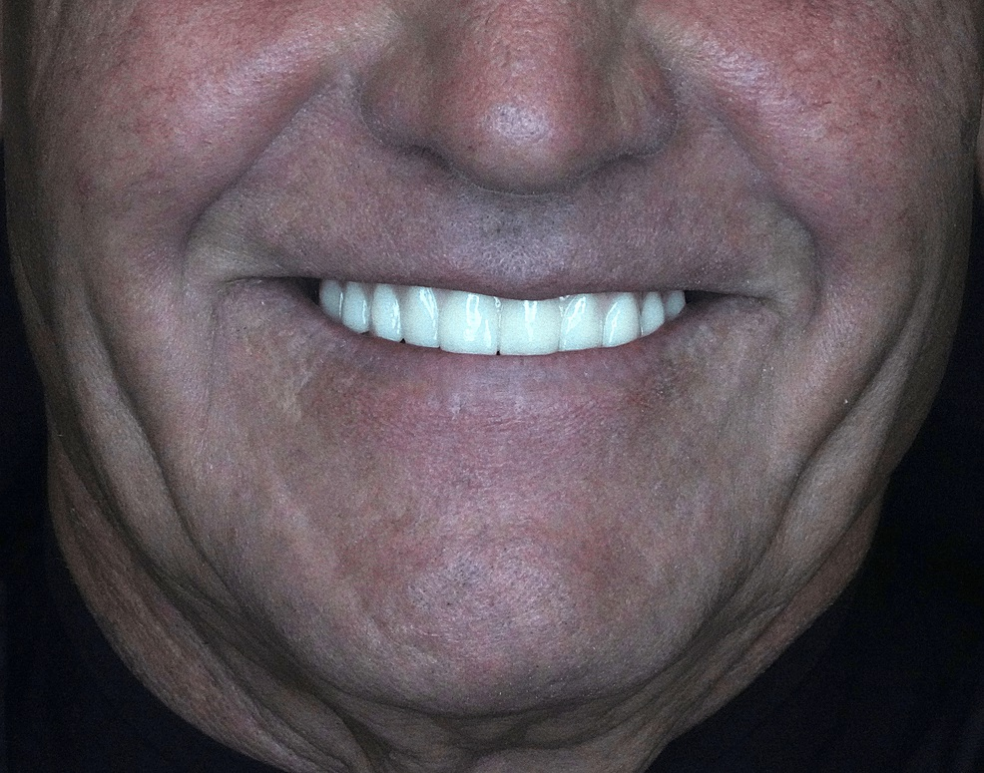
Frontal view of OnX 3D-printed implant-supported prosthesis in mouth.

**Figure 10:**
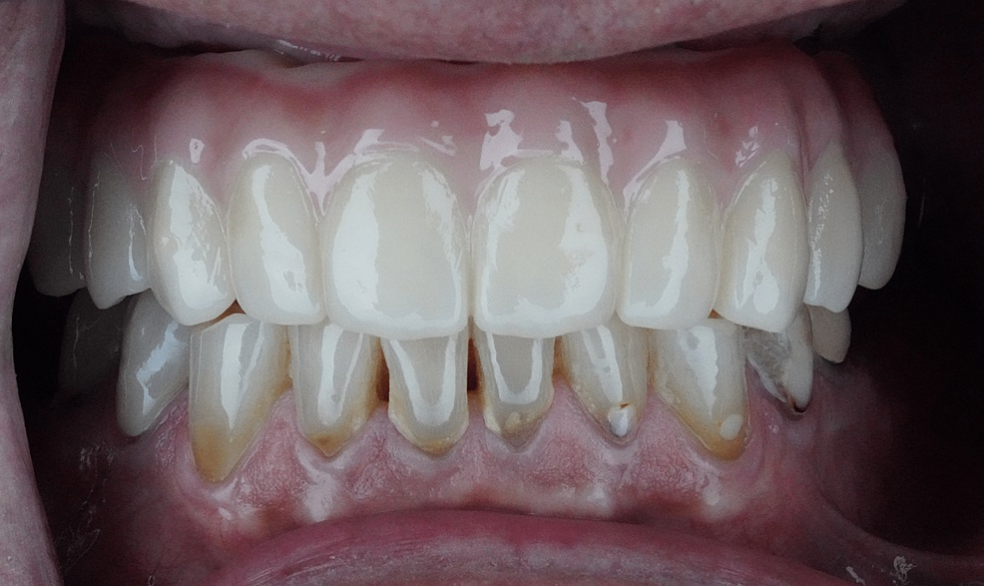
Frontal view in occlusion of highly polished and glazed OnX 3D-printed implant-supported prosthesis in mouth.

The subjects attended follow-up appointments at two months and four months after prosthesis delivery, during which they were advised to promptly notify the clinic of any prosthetic complications. At the end of the four-month period, all patients completed a final OHIP-14 questionnaire as shown in the Appendix section (Figures 9, 10).

Prosthetic survival was determined based on the presence and functionality of the prosthesis at the time of patient evaluation. Complications were defined as any incidents that necessitated prosthetic treatment. The nature and timing of each complication were documented in both the digital patient record and an Excel spreadsheet.

## Results

Prosthetic complications

Over the course of four months, a total of 15 3D-printed full-arch implant prostheses were present and functioning. During the insertion process, all prostheses demonstrated optimal clinical performance, as confirmed by verification models and patient assessments. Throughout the follow-up period, a total of nine prosthetic complications were reported. Among these events, three were classified as catastrophic failures, necessitating the replacement of the same prosthesis using 3D printing technology. On the other hand, nine patients experienced no complications during the evaluation period. The most prevalent issue encountered was prosthesis fracture at the ti-base level, accounting for three out of the nine reported events (Table 1).

**Table 1:** Summary of the number and description of prosthetic complications Ti-base, titanium base.

	15 days	30 days	60 days	120 days
Complication	2 sore areas on tongue. `	1 fractured prosthesis that required repair. Patient reported bruxism.	2 fractured prostheses that required repair.	1 fractured prosthesis that required repair.
Complication		1 catastrophic failure at the level of Ti-base required re-printing.	2 catastrophic failures at the level of Ti-base required re-printing.	

Patient satisfaction

Statistical analysis was performed on the OHIP-14 scores of 15 patients, comprising seven males and eight females. The age range of the participants was between 34 and 79 years, with a mean age of 57.2 years. Among the patients, 12 had natural dentition in the opposing arch, while three were completely edentulous (Tables 2, 3).

**Table 2:** Gender, age, and mean scores of all 15 enrolled patients. ID, identification number; Q1, first OHIP questionnaire; Q2, second OHIP questionnaire; F, female; M, male.

ID	Gender	Age	Total Q1	Total Q2
P1	F	60	29	5
P2	F	45	33	9
P3	F	76	43	3
P4	F	76	43	3
P5	F	70	27	9
P6	M	44	13	4
P7	F	70	24	5
P8	M	61	20	7
P9	M	79	14	4
P10	M	74	51	19
P11	F	65	20	7
P12	M	57	20	6
P13	M	63	38	1
P14	M	52	18	6
P15	M	51	39	7

**Table 3:** Patient characteristics and percentage calculation (N=15)

Characteristic	Sample	Percentage of sample size (N=15)
Gender	Male	47%
	Female	53%
Age	<50 years	27%
	50-65 years	40%
	>65 years	33%
Opposing arch	Natural dentition	80%
	Fixed implant prosthesis	13%
	Complete denture	7%

**Figure 11:**
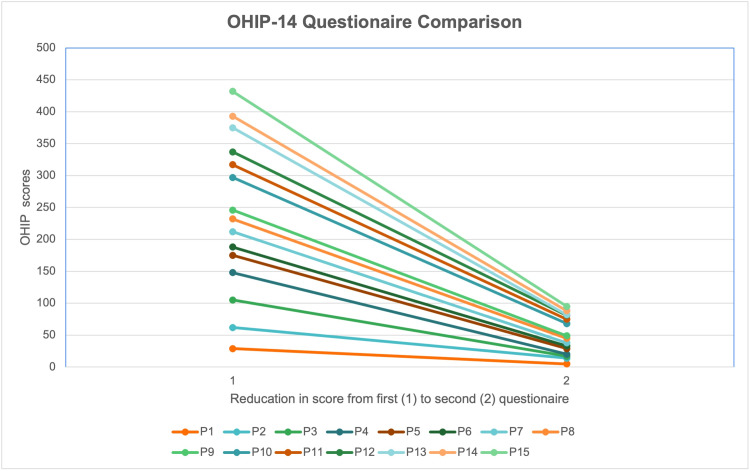
Reduction in mean OHIP-14 scores for all patients from enrollment to four-month postoperative follow-up. P, patient; OHIP-14, Oral Health Impact Profile.

Upon comparing the initial OHIP-14 (Q1) scores with the postoperative OHIP scores (Q2), the statistical analysis revealed no significant difference between males and females in terms of their pre- and postoperative OHIP-14 scores. However, when evaluating patient satisfaction scores between Q1 and Q2, it was observed that, on average, the total OHIP-14 score decreased by approximately 19.5 points from Q1 to Q2. A lower score indicates higher patient satisfaction. This trend was consistent among all patients, as their OHIP-14 scores decreased from the preoperative to postoperative evaluations, suggesting an improvement in their quality of life (Figure 11).

The t-test statistical analysis demonstrated clinically significant differences between Q1 and Q2 (p<0.001), indicating that the 3D-printed OnX prosthesis has a significant positive impact on patients' quality of life and satisfaction.

## Discussion

During this clinical study, a total of nine prosthetic complications were observed, with three of them classified as catastrophic, necessitating the printing of new prostheses within the evaluation period. This study clinically examined the strength of a material used in dental prostheses and identified that all fractures occurred at the titanium base level, where stress concentration is high. Furthermore, it was noted that the CAD software employed for 3D printing does not incorporate a recommended minimum thickness for printed materials. Since 3D-printed materials are still relatively new, research on their properties remains limited and is not integrated into software warnings. While a minimum thickness of 2 mm is generally recommended for other materials, this study suggests that modifying the thickness of the areas surrounding the titanium bases to approximately 4 mm or higher could enhance fracture resistance. Additionally, one patient who experienced a catastrophic failure was found to exhibit signs of bruxism after receiving the 3D-printed prosthesis. Bruxism, a parafunctional habit associated with high stress and loss of proprioception, was identified as a contributing factor. However, it is important to note that such failures can be addressed by generating a second prosthesis from the same 3D file.

Despite the observed complications, there was a significant improvement in OHIP-14 scores and patient satisfaction with the treatment provided, irrespective of age or gender. The Minimal Important Difference (MID), commonly used to interpret mean differences in OHIP-14 scores, considers an average difference of 3 points as clinically meaningful. Thus, the findings of this study, with an average difference of 22.5 points, can be considered clinically significant [15,16].

While 3D printing is not currently suitable for definitive or final dental implant-supported prostheses, it has emerged as a reliable alternative to milling materials. The utilization of OnX 3D printing resin for implant-supported prostheses offers a cost-effective and predictable solution. However, it is crucial to emphasize the importance of proper calibration and post-curing for achieving accuracy and precision. The use of Sprintray Pros S 3D printer in conjunction with Rayware software, developed by the same manufacturer, has been found to be effective in this regard. Additionally, thorough cleaning of the material with 91% isopropyl alcohol and post-curing with the Procure 2 unit (Sprintray, California USA), ensuring optimal temperature, wavelength, and irradiance, are essential steps [17,18]. A controlled environment and consistent workflow are necessary to guarantee predictable outcomes and minimize performance variations among resins. Thus, it is imperative to comprehend and adhere to appropriate post-curing protocols in 3D printing to achieve accurate and reliable results in dental applications [19].

Clinicians may consider exploring options to reduce the overall treatment cost, such as offering a subscription fee for a fixed prosthetic solution, thereby enhancing patients' quality of life. To gain a deeper understanding of the long-term performance and potential complications associated with 3D-printed prostheses, further clinical investigations are warranted.

## Conclusions

Based on the constraints and findings of this study, it can be concluded that the utilization of 3D printing technology with OnX resin represents a highly favorable and promising option for the fabrication of long-term implant-supported temporaries. Clinicians can confidently embrace this innovative approach, as it offers numerous advantages and is associated with significant enhancements in patients' oral health-related quality of life.

The results obtained from this study demonstrate that by employing OnX resin, clinicians can create implant prostheses that contribute to improving patient satisfaction, as well as enhancing the functional and esthetic outcomes. The ability to produce highly accurate prostheses supports the notion that 3D-printed prostheses with OnX resin can be used to withstand the demands of long-term use, offering clinicians and patients a reliable and robust treatment option. Clinicians can confidently embrace this innovative approach as it offers great outcomes for several months and improves oral health-related quality of life while being a cost-effective solution. 

## Appendices

Oral Health Impact Profile questionnaire 

The purpose of the following questionnaire is to measure patient satisfaction with dental treatments provided. Please answer the following questions to the best of your ability:

Codes: 0 never, 1 hardly ever, 2 occasionally, 3 fairly often, 4 very often.  

**Table 4:** Oral Health Impact Profile questionnaire (OHIP-14)

Questions	0	1	2	3	4
1. Have you had trouble pronouncing any words because of problems with your teeth, mouth, or dentures?					
2. Have you felt that your sense of taste has worsened because of problems with your teeth, mouth, or dentures?					
3. Have you had painful aching in your mouth?					
4. Have you found it uncomfortable to eat any foods because of problems with your teeth, mouth, or dentures?					
5. Have you been self-conscious because of your teeth, mouth, or dentures?					
6. Have you felt tense because of problems with your teeth, mouth, or dentures?					
7. Has your diet been unsatisfactory because of problems with your teeth, mouth, or dentures?					
8. Have you had to interrupt meals because of problems with your teeth, mouth, or dentures?					
9. Have you found it difficult to relax because of problems with your teeth, mouth, or dentures?					
10. Have you been a bit embarrassed because of problems with your teeth, mouth, or dentures?					
11. Have you been a bit irritable with other people because of problems with your teeth, mouth, or dentures?					
12. Have you had difficulty doing your usual jobs because of problems with your teeth, mouth, or dentures?					
13.Have you felt that life in general was less satisfying because of problems with your teeth, mouth, or dentures?					
14. Have you been totally unable to function because of problems with your teeth. mouth or dentures?					
